# Overexpression of lncRNA H19/miR-675 promotes tumorigenesis in head and neck squamous cell carcinoma: Erratum

**DOI:** 10.7150/ijms.73148

**Published:** 2022-05-03

**Authors:** Guo-fang Guan, De-jun Zhang, Lian-ji Wen, Ding Xin, Yan Liu, Duo-jiao Yu, Kai Su, Lin Zhu, Ying-yuan Guo, Ke Wang

**Affiliations:** 1 Department of Otolaryngology, Head and Neck Surgery, The Second Hospital of Jilin University, Changchun 130041, P. R. China; 2 Department of Respiratory Medicine, The Second Hospital of Jilin University, Changchun 130041, P. R. China.

Following the publication of this paper, when reviewing the previous research projects, the authors realized that an error was made in the assembly of Figure 4. The uploaded images were selected incorrectly. The authors consider that they made this mistake by selecting a mislabeled folder of images, in which the cells were transfected using a different AntimiR rather that AntimiR-675. After having examined the original data, a corrected version of Figure 4, containing the correct images is presented here. The authors confirm that the replacement of the erroneous images does not affect either the results or the conclusions reported in this paper, and all authors agree to this erratum. The authors sincerely apologize for the error that was introduced during the preparation of this Figure and regret any inconvenience that this mistake has caused.

## Figures and Tables

**Figure 4:**
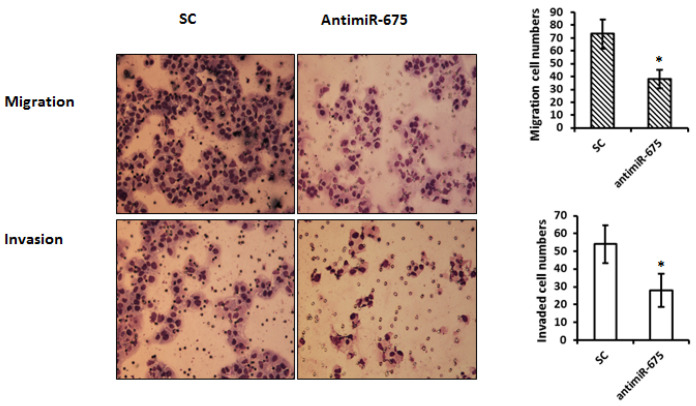
Depletion of miR-675 reduced cell migration and invasion. Top panel--- Representative images of migration assay depict migratory ability after transfection with antimiR-675 compared to scramble control. Bottom panel--- Representative images of invasive assay depict invasiveness after transfection with antimiR-675 compared to scramble control.

